# Effects of Vanadium Microalloying and Intercritical Annealing on Yield Strength–Ductility Trade-Offs of Medium-Manganese Steels

**DOI:** 10.3390/ma16062220

**Published:** 2023-03-10

**Authors:** Nannan Bi, Huaiguang Tang, Zimu Shi, Xingfu Wang, Fusheng Han, Juhua Liang

**Affiliations:** 1 Key Laboratory of Materials Physics, Institute of Solid State Physics, Hefei Institutes of Physical Science, Chinese Academy of Sciences, Hefei 230031, China; 2 Science Island Branch, Graduate School of University of Science and Technology of China, Hefei 230026, China; 3 Anhui Chaohu Foundry Factory Co., Ltd., Hefei 238004, China

**Keywords:** medium-manganese steel, vanadium carbide, strain distribution, mechanical properties

## Abstract

In this paper, we investigate the effects of vanadium on the strength and ductility of medium-manganese steels by analyzing the microstructural evolution and strain hardening rates and performing quantitative calculations. Two significantly different contents of vanadium, 0.05 and 0.5 wt.%, were independently added to model steel (0.12C-10Mn) and annealed at different intercritical temperatures. The results show that higher vanadium addition increases the yield strength but decreases the ductility. The maximum yield strength can increase from 849 MPa to 1063 MPa at low temperatures. The model calculations reveal that this is due to a precipitation strengthening increment of up to 148 MPa and a dislocation strengthening increment of 50 MPa caused by a higher quantity of V_4_C_3_ precipitates. However, the high density of vanadium carbides leads them to easily segregate at grain boundaries or phase interfaces, which prevents strain from uniformly distributing throughout the phases. This results in stress concentrations which cause a high strain hardening rate in the early stages of loading and a delayed transformation-induced plasticity (TRIP) effect. Additionally, the precipitates decrease the austenite proportion and its carbon concentrations, rendering the TRIP effect unsustainable. Accordingly, the ductility of high vanadium steels is relatively low.

## 1. Introduction

Medium-manganese steels with 4–12 wt.% Mn have extensive application potential for a variety of engineering structures, such as autos, ships and trains [1,2,3]. An excellent combination of strength and ductility is one of the most attractive properties of steels [4], as a relatively low yield strength is not desirable for those applications with highly rigid and elastic structures. It is therefore necessary to increase the yield strength while still maintaining a relatively high ductility.

As is well-known, severe plastic deformation, phase transformation or micro-alloying are effective methods used to increase the mechanical properties, particularly the yield strength, of steels [5,6,7]. Huang et al. obtained a yield strength of 0.47C-10Mn-2Al-0.7V steel higher than 2 GPa through multi-pass warm and cold rolling technology [8]. Niu et al. obtained a yield strength of 1221 MPa and a total elongation of 45.3% through multiple cold rolling combined with annealing [9]. Our earlier work also focused on quantifying the relationship between deformation and yield strength [10]. Improvements in mechanical properties are associated with strain-induced martensitic transformation and deformed austenite recrystallization, which lead to reasonable grain distributions and crystalline defects. However, since the enhancement is strongly dependent on large plastic deformation, it is unsuitable or limited for thick materials. In addition, the formation of martensite can essentially reduce the ductility of the material, and cold deformation, working together with complicated heat treatments, causes the processing period to become too long. In contrast, micro-alloying is a straightforward and effective option. Moreover, micro-alloying can greatly increase production efficiency and lower production costs due to the possibility of direct high-temperature deformation.

Many studies have shown that micro-alloying can effectively optimize the yield strength and ductility of steels [11,12,13]. Hamada et al. analyzed variations in VC precipitation with temperature and concluded that VC precipitation strengthening, grain refinement and deformation twining, together, increased the strength and ductility of the steel [14]. Kisko et al. also found that Nb delayed the recrystallization of deformed austenite, resulting in a composite structure of coarse and fine crystals in the steel, thus increasing the strength without reducing the plasticity [15]. Similarly, Allam et al. applied micro-alloy composite additions to X20CrNiMnVN18-5-10 steel to obtain bimodal austenitic grains, resulting in steel with a good strength and ductility [16]. The above studies were on steels with the austenite matrix, where the micro-alloyed elements mainly improved the strength and ductility through grain refinement. However, if there are multiple phases in steels, such as medium-manganese steels with austenite and ferrite laths, the effects of micro-alloying elements on the mechanical properties may become more complex. Tak et al. added different contents of Nb, Ti and V into medium-manganese steel to investigate the effect of micro-alloying [17]. They found that the steel exhibited increased strength but decreased total elongation relative to the base metal. However, Zhang et al. found that microalloying improved the plasticity of the steel through an effective “phase transformation induced plasticity” (TRIP) effect from stable austenite [18]. Other researchers have found that microalloying elements can significantly increase strength but have a weak effect on plasticity [19,20]. The above researchers have presented empirical and experimental findings on how micro-alloying elements affect the mechanical properties of steels. However, there is still debate or insufficient knowledge on the selection of microalloying contents, the temperature dependence of yield strength increase and the effect on ductility. Vanadium is a commonly used microalloying element in medium-manganese steels. Therefore, in this study, we added two different vanadium contents to low-carbon medium-manganese steels to investigate the microstructure evolution and mechanical properties under intercritical annealing. The study also serves as a reference for research on, and applications of, microalloying using medium-manganese steels.

## 2. Experimental Procedure

**Figure 1:**
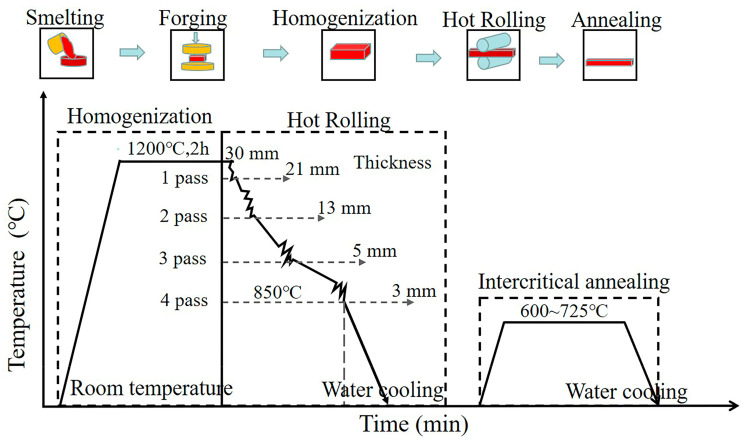
Schematic drawing of the thermomechanical process.

The nominal composition of the medium-manganese steel used in this study is (wt.%) 0.12 C, 10.0 Mn, 1.25 Si and 1.6 Al. The contents of V (wt.%) are 0.5 (named HV) and 0.05 (named LV), respectively. The detailed composition of the steels is shown in Table 1. The alloys were melted in a vacuum induction furnace in an argon atmosphere and cast into ingots. The ingots were then forged into blocks with a length of 600 mm, width of 200 mm and thickness of 30 mm. The forged blocks were subsequently reheated to 1200 °C for 2 h to reach a certain homogenization and then rolled into plates with a thickness of 3 mm, followed by water cooling to room temperature of 25 °C. The total hot rolling reduction ratio was 90%. The hot-rolled plates were intercritically annealed in box furnaces at 600 °C, 650 °C, 675 °C, 700 °C and 725 °C for 30 min and then water-cooled to room temperature. The overall process is shown in Figure 1. To facilitate the identification of the state, the samples were named according to their vanadium contents and annealing temperatures. For example, HV600 represents a high vanadium content and annealed at 600 °C, and vice versa.

The thermodynamic equilibrium phase diagram is from Thermo-calc (database TCFE7). The dog-bone-shaped tensile samples were machined in the rolling direction of the sheets using an electric spark machine with a gauge size 25 mm long, 10 mm wide and 3 mm thick. A material testing system (Instron 3369) was used to measure the mechanical properties of the samples at room temperature and a strain rate of 1 × 10^−3^ s^−1^. Each tensile sample was tested at least three times to ensure repeatability.

The microstructure of the samples was characterized using a SU8020 field emission scanning electron microscope (FE-SEM, Hitachi Company, Tokyo, Japan) and electron backscatter diffraction (EBSD, Oxford Instruments, Oxford, UK) at a voltage of 20 kV and a step size of 0.05. The EBSD data were processed using HKL-Channel 5 software. The phase ratio, carbon content and dislocation density of the austenite were analyzed using an X-ray diffraction apparatus (XRD, Philips Netherlands, Amsterdam, Netherlands, Cu Kα radiation, scan step of 0.01°, scan rate of 0.03° s^−1^, diffraction angle (2θ) of 40–100°). The samples used for microstructure observation were ground and mechanically polished to a diamond size of 0.25 μm and then electrochemically etched with a solution containing 10% perchloric acid and 90% alcohol. The detailed procedure for calculating the dislocation density was reported in a previous study [10]. The volume fraction of austenite was calculated using the integrated intensities of the (200)_α_ and (211)_α_ peaks, as well as the (220)_γ_ and (311)_γ_ peaks, combined using the following formula [21]:

(1) $$V_{\gamma }+V_{\alpha }=1$$

(2) $$V_{\gamma }=1.4I_{\gamma }/\left(I_{\alpha }+1.4I_{\gamma }\right)$$

where $V_{\gamma }$ and $V_{\alpha }$ are the volume fractions of austenite and ferrite, respectively, and $I_{\gamma }$ and $I_{\alpha }$ are the integrated peak intensities of the austenite and ferrite phases, respectively.

The nanostructure of the samples was observed using a JEM-2010F transmission electron microscope (TEM, JEOL Ltd, Tokyo, Japan). The samples used in the TEM observation were mechanically polished to a thickness of 60 μm, punched to obtain disc-like specimens with a diameter of 3 mm and, finally, electro-polished in a solution of CH_3_COOH (90%) and HClO_4_ (10%) using a twin-jet electro-polishing apparatus to obtain thin foil specimens.

## 3. Results

### 3.1. Thermo-Calc Analysis

**Figure 2:**
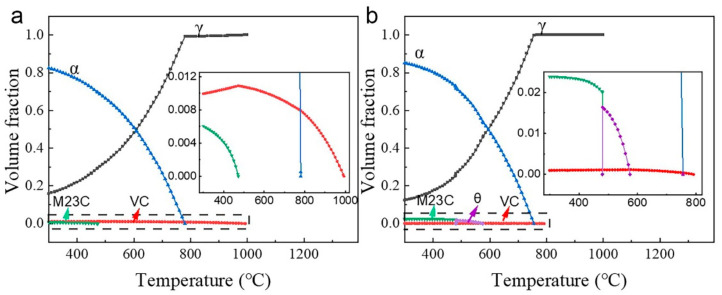
Equilibrium phase diagrams of (**a**) HV steel and (**b**) LV steel. The insets are the enlarged images in the dashed boxes of the corresponding figures.

Figure 2 shows thermodynamic equilibrium phase diagrams for HV and LV steels. According to the figure, HV steels reach full austenitization at 840 °C, while LV steels do so at 817 °C. HV steels also have a lower proportion of austenite at the same temperature, ranging from 600 to 800 °C. This indicates that the vanadium content significantly influences the growth of austenite. In addition, no cementite (θ phase) precipitates in HV steels at low temperatures, which indicates that the 0.481% V element can completely prevent cementite from forming. When the temperature exceeds 600 °C, only one carbide, i.e., vanadium carbide, is left in both steels, as the other carbides are gradually dissolved. These results provided a reference for subsequent microstructural analysis.

### 3.2. Microstructures

**Figure 3:**
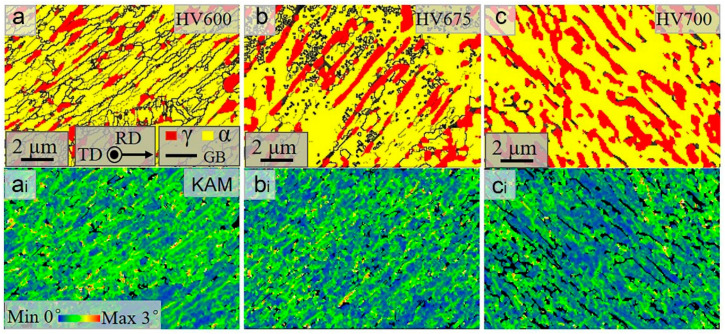
IQ phase maps for HV steel samples (**a**–**c**) and KAM maps for HV steel samples (**a_i_**–**c_i_**) annealed at different temperatures.

**Figure 4:**
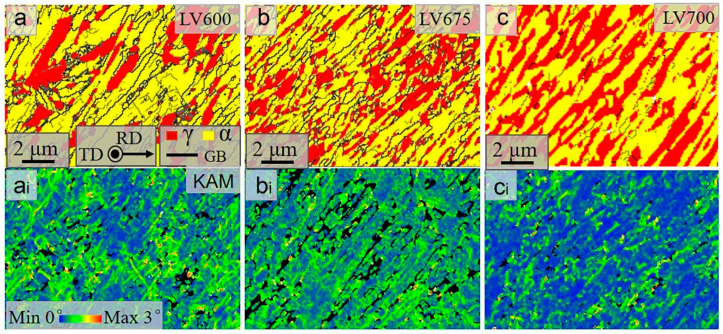
IQ phase maps for LV steel samples (**a**–**c**) and KAM maps for LV steel samples (**a_i_**–**c_i_**) annealed at different temperatures.

The typically annealed microstructures of lath ferrite–austenite dual-phase structures at 600 °C, 675 °C and 700 °C are shown in Figure 3 and Figure 4, in which yellow refers to ferrite and red refers to austenite. The ferrite lath width is less than 250 nm, inheriting the morphology of the hot-rolled martensitic matrix. As the annealing temperature rises, the austenite fraction and size in both steels increase, as shown in Figure 3 and Figure 4. Interestingly, the volume fraction of austenite in HV steels is always lower than that in LV steels at the same temperature. This is because more VC precipitates in HV steels, which reduces the carbon content of austenite [20], thereby increasing the complete austenitization temperature. Furthermore, the presence of VC particles pins the phase interface and prevents the growth of austenite [22,23]. The Kernel average misorientation (KAM) maps shown here represent the density of lattice defects in the samples, including grain boundaries, phase boundaries and dislocations. Figure 3(a_i_–c_i_) and Figure 4(a_i_–c_i_) display the KAM maps corresponding to Figure 3a–c and Figure 4a–c, respectively, in which the green color represents high lattice defects. This shows that the KAM values decrease with increasing temperature. At 600 °C and 675 °C, the KAM values of the two steels are similar. However, above 700 °C, KAM values for HV steels are significantly lower than those for LV steels.

**Figure 5:**
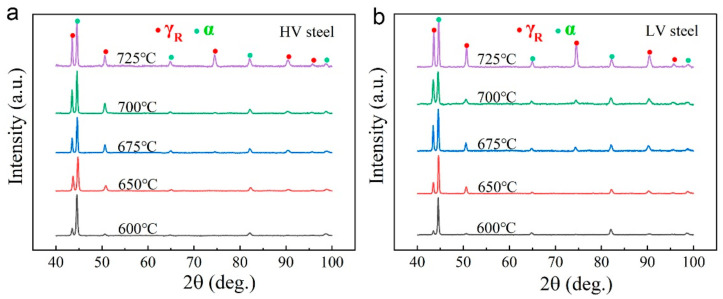
XRD patterns of samples annealed at different temperatures: (**a**) HV steels; (**b**) LV steels. The diffraction peaks corresponding to the red dots refer to austenite, while green dots refer to ferrite.

Figure 5 shows the XRD patterns of all the samples, from which the austenite fraction and dislocation density of the ferrite were calculated, as shown in Table 2. As the annealing temperature increased, the austenite fractions of both the HV and LV steels continuously increased, while ferrite exhibited the opposite behavior, indicating that austenite undergoes a reverse-phase transformation. Compared with HV steels, LV steels exhibited not only a higher austenite fraction but also a higher austenite growth rate, which is consistent with the austenite trend shown in Figure 3 and Figure 4. The dislocation density of both steels decreased with increasing temperature, since high temperatures result in dislocation recovery and annihilation. Additionally, the HV steels always had a higher dislocation density than the LV steels at the same temperature, which is evidently also the result of carbide pegging dislocations [24,25].

### 3.3. Mechanical Properties

**Figure 6:**
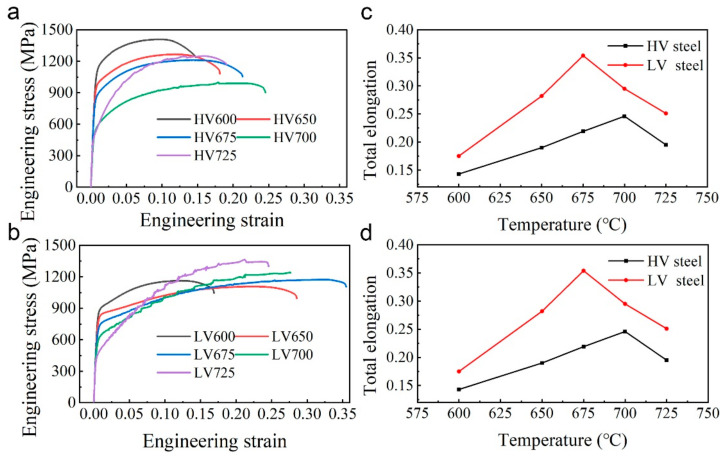
Mechanical properties of HV and LV samples at different annealing temperatures: engineering stress–strain curves of (**a**) HV steels and (**b**) LV steels; (**c**) yield strength curves and (**d**) total elongation curves.

The mechanical properties of the samples are shown in Figure 6. Figure 6a,b shows that the HV steel samples had a yield strength ranging from 400 MPa to 1100 MPa, whereas that of the LV steel samples ranged from 400 MPa to 800 MPa, depending on the annealing temperature. The yield strength of HV steels was always higher than that of LV steels at the same temperature, as shown in Figure 6c. However, the yield strength and differences in yield strength between the two steels showed a similar downward trend with increasing temperatures. High vanadium addition was beneficial for increasing the yield strength, while total elongation showed a different tendency. As shown in Figure 6d, the total elongation first increased until the peak and then decreased. The largest elongation of LV steel was 35% at 675 °C, which was higher than that of HV steel, being 25% at 700 °C. Moreover, the total elongation of LV steels was always higher than HV samples at the same temperature. In summary, high vanadium addition can be favorable for strength but unfavorable for ductility, an observation which is consistent with some other researchers’ reports [26]. Among others, HV675 and LV650 showed the best comprehensive mechanical properties, with a yield strength above 750 MPa and total elongation of over 20%, respectively, as shown in Figure 6c,d.

### 3.4. Nanoscale Morphology

**Figure 7:**
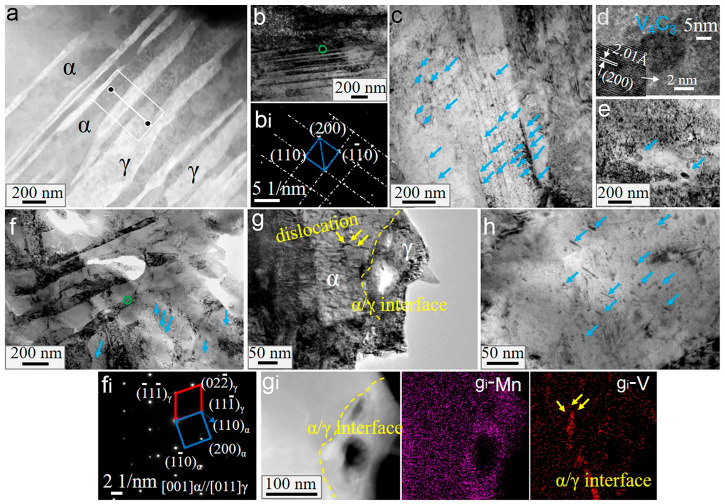
TEM micrographs of HV600 (**a**–**e**) and HV700 (**f**–**h**): (**a**) EDXS line profile analysis; (**b**) ultrafine ferrites; (**c**) carbides around the interfaces; (**b_i_**) the selected area diffraction (SADP) in the green circle in (**b**); (**d**) high-resolution TEM (HRTEM) images of carbide; (**e**) dislocations and carbides intertwining; (**f**,**g**) the bright field images; (**f_i_**) and (**g_i_**) SADP of (**f**) and EDXS surface analysis of (**g**); g_i_-Mn and g_i_-V refer to the distribution of Mn and V elements after surface analysis of g_i_, respectively; (**h**) distribution of carbides. The blue arrows refer to the carbide, the yellow arrows refer to dislocation, and the yellow dashed line refers to the phase interface in the figure.

**Figure 8:**
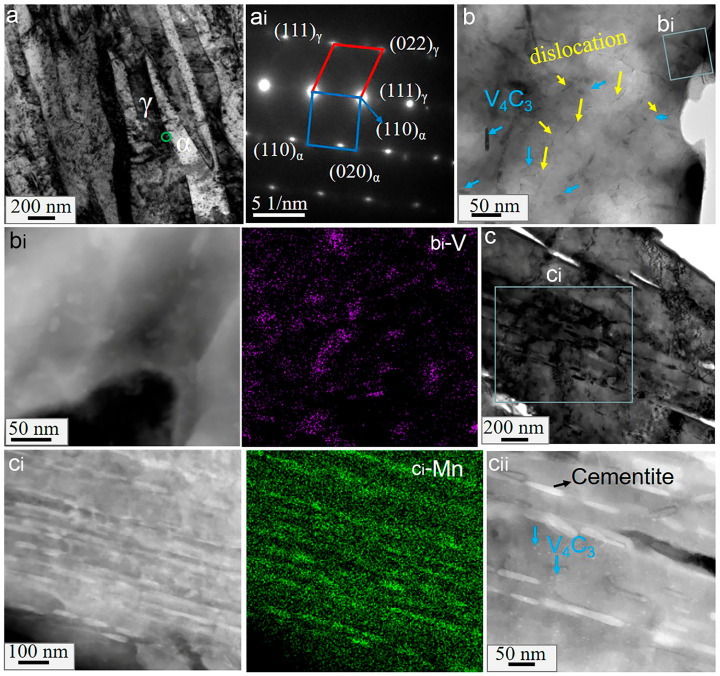
TEM micrographs of sample LV600: (**a**) the bright field images; (**b**,**c**) the distribution of carbides, where (**a_i_**), (**b_i_**) and (**c_i_**) are the corresponding SADP in (**a**); EDXS surface analysis for (**b**) and (**c**), respectively, where (**c_ii_**) is a magnification of (**c_i_**); b_i_-Mn and c_i_-V refer to the distribution of V and Mn elements after surface analysis of b_i_ and c_i_, respectively. The black arrow refers to cementite.

**Figure 9:**
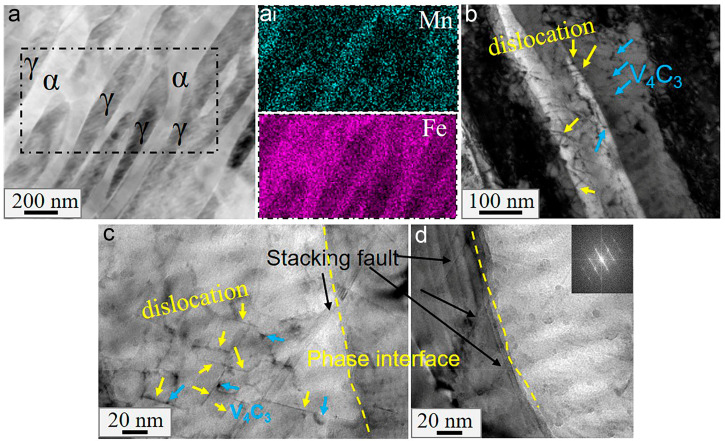
TEM micrographs of sample LV700: (**a**) the dark field images, where (**a_i_**) is the EDXS surface analysis of (**a**); (**b**) the distribution of dislocations; (**c**) microstructure near the phase interface; (**d**) the distribution of stacking faults, where the inset is the corresponding fast Fourier transform.

The interaction between carbides, dislocations, grain boundaries, etc., is the primary reason for the differences in mechanical properties between HV steels and LV steels. To clarify the action mechanism of vanadium carbides, TEM observations of the samples of the two steels were performed after annealing at 600 °C and 700 °C, respectively, as shown in Figure 7, Figure 8 and Figure 9.

Figure 7a–e depicts the characterization of sample HV600, while Figure 7f–h depicts that of sample HV700. In sample HV600, the austenite lath or film is 50–250 nm in width and uniformly distributed within the lath ferrite, as shown in Figure 7a, which can better relieve stress concentration [27]. From the inset in Figure 7a, we can see that the manganese content in the austenite film increased during annealing due to its high inherent solubility. The element redistribution increases the carbon content of austenite and thus increases its stability, favoring high plasticity through the so-called TRIP effect. Ultrafine ferrite also exists in the HV600 sample, as shown in Figure 7(b,b_i_), which is a typical microstructure formed during the intercritical annealing of medium-manganese steels [28]. As expected, the HV steels had a large number of spherical or fibrous carbides distributed not only within the grains but also at the phase interface, as shown in Figure 7c. Figure 7d shows that these carbides were V_4_C_3_. Most of the fibrous carbides were parallel to the phase interface and had a mean size of 5 nm in width and 35 nm in length, while the diameters of the spherical carbides ranged from 5 to 15 nm. Whether these carbides were fibrous or spherical, they have been observed in our previous studies [10]. When the annealing temperature was relatively low, many dislocations remained and intertwined with the carbide particles, as shown in Figure 7e. When the annealing temperature was high, for example, at 700 °C, most carbides were still segregated at the interfaces, as shown in Figure 7f. The surface analysis revealed high vanadium segregation at the ferrite–austenite interface and dislocations near the interface. It can be seen from Figure 7(f_i_) that the ferrite and austenite have the following relationship: ${\left(11\bar{1}\right)}_{\gamma }$//${\left(110\right)}_{\alpha }$ and ${\left[001\right]}_{\alpha }$//${\left[011\right]}_{\gamma }$, which is reported to facilitate the segregation of carbides [29].

Similar to HV steels, LV steels also have alternately distributed austenitic and ferritic laths, as shown in Figure 8(a,a_i_). Figure 8(b,b_i_) show that vanadium carbide particles are mainly distributed in the ferrite, and their number is less than that of HV steels. Additionally, there are visible dislocation lines surrounding the vanadium carbides, indicating that vanadium carbides have a pinning effect on the dislocations. From Figure 8c, it can be seen that the LV600 sample not only contained fine vanadium carbides in the grains (Figure 8(c_ii_)) but also a large amount of cementite, with a diameter ranging from 50 to 100 nm. As shown in Figure 8(c_i_,c_ii_), the cementite surrounded by dislocations was parallel with the grain boundaries, adopting the position where vanadium carbide would otherwise segregate in HV steels.

When the temperature increased to 700 °C, the cementite disappeared, as shown in Figure 9(a,a_i_). Figure 9b shows many dislocation lines and vanadium carbides distributed throughout the austenite and ferrite laths. Notably, some dislocation lines were observed across the phase interface. Compared to HV steels, no obvious interface segregation was observed in LV700. Interestingly, several stacking faults were observed at the phase interface in the LV700 sample, as shown in Figure 9c,d.

## 4. Discussion

### 4.1. Effect of Vanadium on the Microstructures

**Figure 10:**
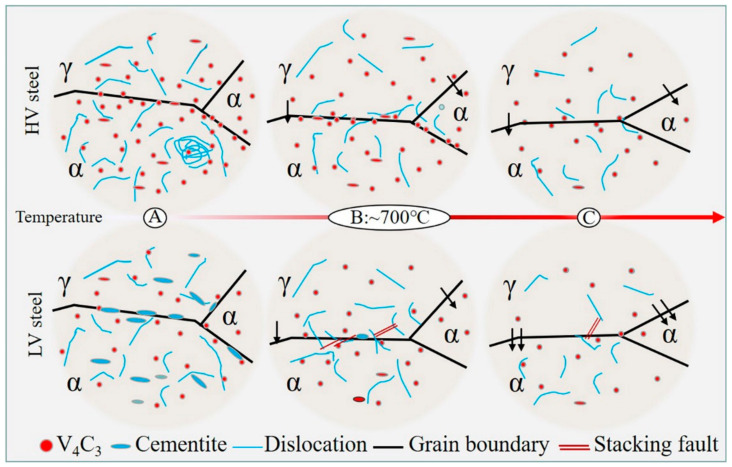
Schematic diagram of the phase interface evolution of HV steels and LV steels with increasing temperature. Stage A: ~600 °C, stage B: ~700 °C, stage C: over 700 °C or more.

As mentioned above, different vanadium contents lead to different types, distributions and densities of carbides in steels, which affect the movement of interfaces and dislocations and, therefore, result in clearly different strengths and degrees of ductility of steels. Based on the microstructure characterizations shown above, a schematic diagram was drawn up to summarize the microstructure evolution around the phase interface, as shown in Figure 10. Alloying elements, such as carbon, are redistributed during intercritical annealing. As a strong carbide former, vanadium preferentially combines with carbon to form carbides, preventing its diffusion into austenite. This leads to a lower carbon content of austenite in HV steels than in LV steels [20]. According to Figure 7 and Figure 8 and Thermo-calc, LV steels exhibit cementite at low temperatures. This is mainly because the lower amount of vanadium (0.05 wt.%) in LV steels can only partially consume the carbon, leaving the rest to form cementite, which fills the phase interface, as shown in Figure 8(c_ii_) and stage A in Figure 10. As the temperature increases to 700 °C, the cementite is completely dissolved due to instability, leaving only the stable vanadium carbide. The dissolution of cementite supports the growth of austenite during intercritical annealing. It can serve as an austenite nucleation site, and it can also provide a carbon source for the growth of austenite [30]. As a result, the austenite fraction of LV steels increases more quickly. In contrast, due to the higher density and harder solubility of vanadium carbide, carbides still segregate at the grain boundaries in HV steels until reaching 700 °C, as shown in stage B of Figure 10, causing a significant solute drag effect that slows the migration rate of the phase interface [20,22,31]. Therefore, the fraction of austenite does not change significantly until about 700 °C, as shown in Table 2. When the temperature continues to rise, as shown in stage C, the dissolution of vanadium carbide accelerates, resulting in a less pronounced segregation and dragging effect and, therefore, significant increases in the carbon concentration and the fraction of austenite. In summary, HV steels have a complex interface structure, with segregated vanadium carbide and pinned dislocations around the interface. In LV steels, however, the cementite is larger and has a weaker pegging effect on the phase interface than vanadium carbide, while at 700 °C, the carbides gradually dissolve, reducing their density, and there is no significant segregation at the phase interface. The phase interface is relatively simple and pure. When the dislocation moves to the phase interface, it can easily to lead to sliding along the interface or even generate new dislocations on the austenite side, leading to the formation of lattice defects near the interface, as shown in Figure 9 and stages B and C of Figure 10 [31].

### 4.2. Effects of Vanadium on the Strain Hardening Rate and Dynamic Strain Ageing

**Figure 11:**
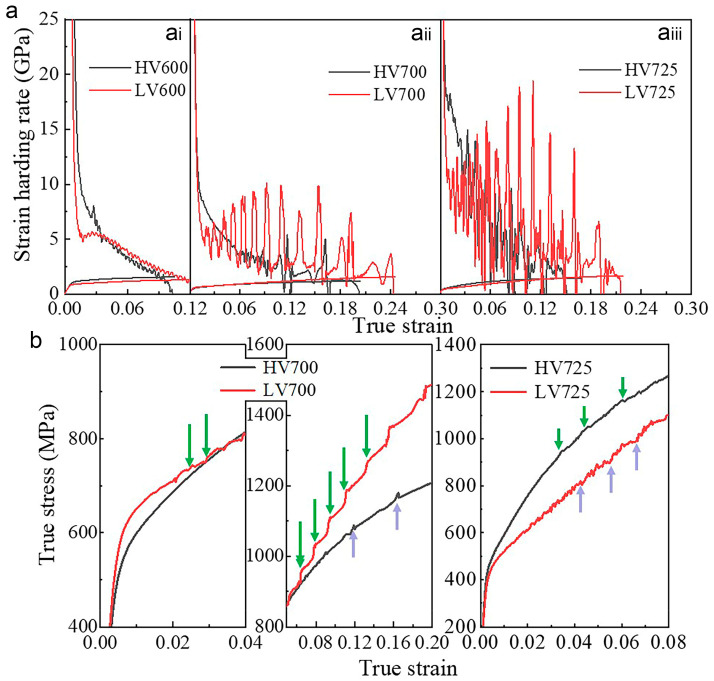
(**a**) True stress–strain curves and corresponding stain hardening rate curves of HV and LV steels annealed at (**a**) 600 °C, (**a_ii_**) 700 °C and (**a_iii_**) 725 °C, respectively; (**b**) enlarged true stress-strain curves in (**a_ii_**) and (**a_iii_**), respectively. The green arrows refer to the serrations on the SHR curves for HV steels, while the purple ones refer to those for LV steels.

Figure 11 shows the true stress–strain and strain hardening rate (SHR) curves of HV and LV steels annealed at 600 °C, 700 °C and 725 °C for 30 min, respectively. As is well-known, strains are mainly modified by dislocation slip in the early stages of deformation, whereas later stresses are mainly released through the combined effect of precipitates, dislocations and the TRIP effect which, together, determine the plasticity of the material [32].

In HV steels, the segregated carbides strongly pin the dislocations and interfaces, forming a complex interface, which is reported to affect the ease of dislocation transfer from one phase to another [33]. It is difficult for dislocations to slip across the segregated interface into the austenite, meaning that the stress is not released into the austenite but concentrated in the ferrite [34]. In general, the accumulation of dislocations often results in high stress concentrations near the phase interface. When the stress concentration approaches a threshold, dislocations will nucleate on the austenite side, forming new slip dislocations. Then, it is possible to release the stress concentration. Moreover, the stress concentrations become relaxed once the barriers collapse. However, the segregated vanadium carbide pins the dislocations and phase interface, raising the threshold and thus delaying the formation and slip of dislocations, whereas in LV steels, especially up to 700 °C, the stress can more easily cross the interface and disperse into the austenite, thus activating dislocation slip in the austenite and even phase change to martensite, i.e., the TRIP effect. Figure 11a shows that the SHR of HV steels is higher than that of LV steels during the early stages of strain. This is due to the increased dislocation slip resistance caused by vanadium carbide, which has been proven to affect the SHR in Fe–Mn–Al–C lightweight steel [35]. The SHR curves of the LV700 sample already start to fluctuate before 0.03 strain, as shown in Figure 11(a_ii_,a_iii_), which is associated with the discontinuous strain-induced martensitic transformation of austenite [36]. This shows that the phase transformation in LV steels can be triggered by relatively low strains. In contrast, the SHR of the HV700 sample does not begin to fluctuate until the strain exceeds 0.8. In addition, due to the lower carbon content in austenite and its volume fraction, the TRIP effect is terminated earlier, and the SHR curve stops earlier. As the temperature rises to 725 °C, the SHRs of both HV725 and LV725 start to fluctuate very early on, as shown in Figure 11(a_iii_).

Dynamic strain ageing (DSA) is a common phenomenon in medium-manganese steels, presenting a typical sawtooth flow of the stress–strain curve, as shown in Figure 11b. There are conflicting theories about the formation mechanism of DSA in manganese steel [37,38], some of which are related to the interaction between solute atoms and dislocations. Lee et al. found that aluminum affects the activity and diffusion rate of carbon in austenite in high-Mn TWIP steel, thus changing the DSA of steel [37]. By comparison, Tak et al. concluded that vanadium carbide affects the carbon content of ferrite by affecting the DSA of steel [20]. Based on these and previous analyses, we infer that vanadium affects the DSA of steel by altering the carbon content and dislocation density, as described below. At low temperatures, the effects of solutes and dislocations are negligible because of the large amounts of vanadium carbide or cementite precipitate, lowering the carbon activity in the matrix. As the temperature increases, the carbides gradually dissolve, with cementite dissolving faster than vanadium carbide, thus increasing the matrix’s carbon concentration. Meanwhile, in LV steels, the strain can more easily cross the interface to promote the formation of dislocations or defects in the austenite (Figure 9). Therefore, the interaction between carbon and dislocations is relatively intense in LV steels, leading to a more pronounced DSA, as seen in Figure 11b. The DSA facilitates the separation of dislocations and relieves stress concentration at the grain boundaries [39].

### 4.3. Effect of Vanadium on the Yield Strength

The contributions of alloying elements to yield strength mainly include solid solution strengthening, precipitation strengthening, grain refinement and dislocation strengthening. To explore the effect of vanadium on the yield strength of medium-manganese steels, here, we consider the difference in the total yield strength between HV and LV steels. In this experiment, we assumed that the difference in yield strength caused by vanadium carbide is mainly due to precipitation strengthening and dislocation strengthening in the ferrite, ignoring the roles of solid solution strengthening and grain boundary strengthening at the same temperature. The specific model formula is as follows [40]:

(3) $$\Delta \sigma =\Delta \sigma_{HL}^{dis}+\Delta \sigma_{HL}^{pre}$$

where Δσ, $\Delta \sigma_{HL}^{dis}$ and $\Delta \sigma_{HL}^{pre}$ are the total yield strength difference, dislocation strengthening difference and precipitation strengthening difference of HV and LV steels, respectively. The precipitation strengthening difference is as follows [41]:

(4) $$\Delta \sigma_{HL}^{pre}=\sigma_{H}^{pre}-\sigma_{L}^{pre}$$

(5) $$\sigma_{i}^{pre}=8.995\times 10^{3}\left(f_{i}^{1/2}/d_{i}\right)\ln\left(2.417d_{i}\right)$$

where $\sigma_{i}^{pre}$ is the precipitation strengthening of *i* steel, *i* refers to HV steels (H) or LV steels (L), $d_{i}$ is the diameter of the vanadium carbide particles, and $f_{i}$ is the volume fraction of vanadium carbide particles derived from Thermal-calc (Figure 2). The diameter of vanadium carbide particles depends on the minimum diameter and the ageing rate [41]. The ageing rate, at 600–700 °C, does not change significantly; thus, it is considered that the average particle size of vanadium carbide is almost unchanged in the range of 600–675 °C, which is equal to 10 nm, while at 725 °C, it is equal to 15 nm at 700 °C. Similarly, the precipitation strengthening difference ($\sigma_{i}^{dis}$) is obtained according to the following formula [42]:

(6) $$\Delta \sigma_{HL}^{dis}=\sigma_{H}^{dis}-\sigma_{L}^{dis}$$

(7) $$\sigma_{i}^{dis}=M_{i}\alpha_{i}G_{i}b_{i}\sqrt{\rho_{i}}$$

where $\sigma_{i}^{dis}$ is the precipitation strengthening of *i* steel; $M_{i}$ is the Taylor factor, 3.06; $\alpha_{i}$ is a constant; $G_{i}$ is the shear modulus, 80 GPa; $b_{i}$ is the Burgers vector, 0.248 nm; and $\rho_{i}$ is the dislocation density of ferrite, as shown in Table 2.

**Figure 12:**
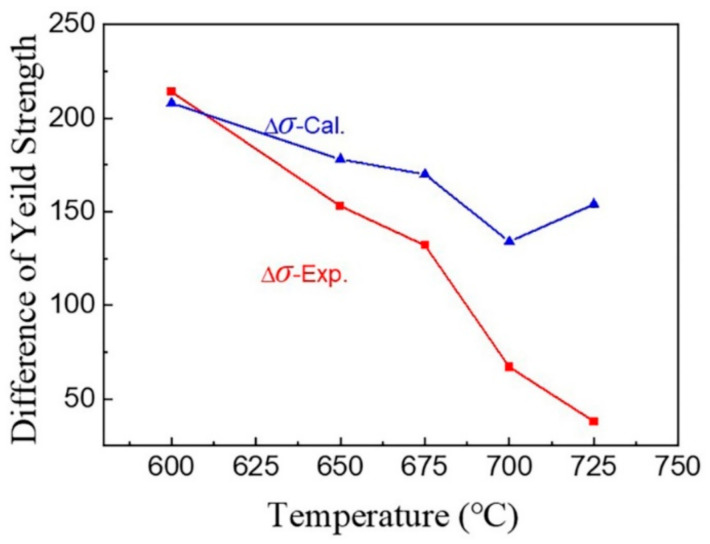
Calculated and experimental yield strength differences between HV and LV steels.

The calculated yield strength differences between HV steels and LV steels are listed in Table 3. The total yield strength difference, together with the experimental value, is plotted in Figure 12. It is shown that the yield strength difference between the two steels mostly originates from the precipitation strengthening caused by microalloying. At low temperatures, the yield strength difference between the two steels is up to 208 MPa. This is mostly due to the precipitation strengthening difference of about 148 MPa and the dislocation strengthening difference of about 60 MPa, caused by the intense pinning of the precipitated particles. As the temperature increases, the yield strength difference decreases, indicating that the strengthening capacity of the vanadium element is diminished at high temperatures. Additionally, the calculated and experimental values are closer at low temperatures, whereas the deviation increases as the intercritical annealing temperature rises. This may be related to the assumed larger grain size of vanadium carbide, the change in grain size as the temperature increases or the increasing rate of the austenite fraction at higher temperatures. 

## 5. Conclusions

The effects of vanadium addition and intercritical annealing on the microstructure and mechanical properties of low-carbon medium-manganese steels were investigated in the present study. The conclusions are as follows.

1. A high vanadium content leads to a high density of vanadium carbides, which strongly pins the dislocations and grain boundaries in the matrix. The dragging effect caused by the pinning slows down the growth of austenite during intercritical annealing, and the reduction in the solid carbon content caused by high vanadium alloying causes the austenite fraction of HV steel to always be smaller than that of LV steel at the same temperature.
2. The formation of vanadium carbide is preferential to that of cementite. During low-temperature intercritical annealing, the addition of high vanadium prevents the formation of brittle cementite and thus favors the mechanical properties. At 600 °C, HV steels have an obvious yield strength increment of almost 200 MPa, which is mainly due to high-density vanadium carbide precipitation and pinned dislocation. However, both the yield strength and its increment clearly decrease with increasing temperature.
3. Vanadium carbide has a tendency to segregate at the phase interface in HV steels at 700 °C. This complex interface is not conducive to the redistribution of strain within the grain, meaning that it is easy to produce stress concentration and delay the occurrence of the TRIP effect. Therefore, excessive vanadium addiction is harmful to strain coordination during the plastic deformation of medium-manganese steel.

## Figures and Tables

**Table 1:** The analyzed chemical compositions of the present steels.

Steels	Element and Content (wt.%)							
	C	Si	Mn	Al	V	P	S	Fe
HV	0.13	1.26	9.70	1.58	0.481	0.029	0.006	Bal.
LV	0.12	1.26	9.90	1.65	0.051	0.021	0.003	Bal.

**Table 2:** The volume fraction of retained austenite and dislocation density of ferrite calculated from the XRD patterns (γ_R_: retained austenite; α: ferrite).

Samples	Volume Fraction (vol.%)		Dislocation Density in α (m^−2^)
	γ_R_	α	
HV600	28.6	70.4	3.621 × 10^14^
HV650	34.6	64.6	3.094 × 10^14^
HV675	42.4	56.6	2.620 × 10^14^
HV700	47.0	52.2	2.356 × 10^14^
HV725	56.1	42.1	2.240 × 10^14^
LV600	36.0	64.0	2.632 × 10^14^
LV650	39.8	60.2	2.595 × 10^14^
LV675	52.3	47.4	2.287 × 10^14^
LV700	58.3	41.7	2.073 × 10^14^
LV725	69.0	31.0	1.853 × 10^14^

**Table 3:** Differences in yield strength according to dislocation and precipitation.

Temperature (°C)	$\Delta \sigma_{HL}^{dis}$ (MPa)	$\Delta \sigma_{HL}^{pre}$ (MPa)
600	60	148
650	32	146
675	23	147
700	20	114
725	37	117

## Data Availability

The data that support the findings of this study are available from the corresponding authors upon reasonable request.

## References

[1] Lee Y.K., Han J. (2015). Current opinion in medium manganese steel. Mater. Sci. Technol..

[2] Bartlett L., Van Aken D. (2014). High Manganese and Aluminum Steels for the Military and Transportation Industry. JOM.

[3] Ma Y. (2017). Medium-manganese steels processed by austenite-reverted-transformation annealing for automotive applications. Mater. Sci. Technol..

[4] Wei Y.J., Li Y.Q., Zhu L.C., Liu Y., Lei X.Q., Wang G., Wu Y.X., Mi Z.L., Liu J.B., Wang H.T. (2014). Evading the strength- ductility trade-off dilemma in steel through gradient hierarchical nanotwins. Nat. Commun..

[5] Hu B., He B.B., Cheng G.J., Yen H.W., Huang M.X., Luo H.W. (2019). Super-high-strength and formable medium Mn steel manufactured by warm rolling process. Acta Mater..

[6] Yong G., Han D. Review of applications of vanadium in steels. Proceedings of the International Seminar on Production and Application of High Strength Seismic Grade Rebar Containing Vanadium.

[7] Li Z.C., Zhang X.T., Mou Y.J., Misra R.D.K., He L.F., Li H.P. (2019). The impact of intercritical annealing in conjunction with warm deformation process on microstructure, mechanical properties and TRIP effect in medium-Mn TRIP steels. Mater. Sci. Eng. A.

[8] He B.B., Hu B., Yen H.W., Cheng G.J., Wang Z.K., Luo H.W., Huang M.X. (2017). High dislocation density-induced large ductility in deformed and partitioned steels. Science.

[9] Niu G., Wu H.B., Zhang D., Gong N., Tang D. (2018). Heterogeneous nano/ultrafine-grained medium Mn austenitic stainless steel with high strength and ductility. Mater. Sci. Eng. A.

[10] Bi N.N., Liang J.H., Wang X.F., Kang T., Han F.S. (2022). Microstructure evolution and yield strength improvement of a low carbon medium manganese steel experienced intercritical annealing, pre-straining and tempering. Mater. Charact..

[11] Han Y., Shi J., Xu L., Cao W.Q., Dong H. (2011). TiC precipitation induced effect on microstructure and mechanical properties in low carbon medium manganese steel. Mater. Sci. Eng. A.

[12] Lee S., Estrin Y., De Cooman B.C. (2013). Constitutive Modeling of the Mechanical Properties of V-added Medium Manganese TRIP Steel. Metall. Mater. Trans. A.

[13] Pan H.J., Ding H., Cai M.H. (2018). Microstructural evolution and precipitation behavior of the warm-rolled medium Mn steels containing Nb or Nb-Mo during intercritical annealing. Mater. Sci. Eng. A.

[14] Hamada A., Kömi J. (2018). Effect of microstructure on mechanical properties of a novel high-Mn TWIP stainless steel bearing vanadium. Mater. Sci. Eng. A.

[15] Kisko A., Hamada A., Talonen J., Porter D., Karjalainen L. (2016). Effects of reversion and recrystallization on microstructure and mechanical properties of Nb-alloyed low-Ni high-Mn austenitic stainless steels. Mater. Sci. Eng. A.

[16] Allam T., Guo X., Sevsek S., Lipińska-Chwałek M., Hamada A., Ahmed E., Bleck W. (2019). Development of a Cr-Ni-VN medium manganese steel with balanced mechanical and corrosion properties. Metals.

[17] Park T.M., Kim H.-J., Um H.Y., Goo N.H., Han J. (2020). The possibility of enhanced hydrogen embrittlement resistance of medium-Mn steels by addition of micro-alloying elements. Mater. Charact..

[18] Zhang G.-T., Zhu N.-Q., Sun B.-W., Zhao Z.-Z., Zheng Z.-W., Tang D., Li L. (2021). Effect of V Addition on Microstructure and Mechanical Properties in C–Mn–Si Steels after Quenching and Partitioning Processes. Metals.

[19] Zhang K., Liu P., Li W., Guo Z.H., Rong Y.H. (2015). High Strength-Ductility Nb-microalloyed Low Martensitic Carbon Steel: Novel Process and Mechanism. Acta. Metall. Sin.-Engl..

[20] Park T.M., Jeong M.S., Jung C., Choi W.S., Choi P.-P., Han J. (2021). Improved strength of a medium-Mn steel by V addition without sacrificing ductility. Mater. Sci. Eng. A.

[21] Song Y., Li X., Rong L., Li Y. (2011). The influence of tempering temperature on the reversed austenite formation and tensile properties in Fe–13% Cr–4% Ni–Mo low carbon martensite stainless steels. Mater. Sci. Eng. A.

[22] Santofimia M.J., Zhao L., Sietsma J. (2008). Model for the interaction between interface migration and carbon diffusion during annealing of martensite–austenite microstructures in steels. Scripta Mater..

[23] Varanasi R.S., Lipinska-Chwalek M., Mayer J., Gault B., Ponge D. (2022). Mechanisms of austenite growth during intercritical annealing in medium manganese steels. Scripta Mater..

[24] Andrade H., Akben M., Jonas J. (1983). Effect of molybdenum, niobium, and vanadium on static recovery and recrystallization and on solute strengthening in microalloyed steels. Metall. Trans. A.

[25] Ollilainen V., Kasprzak W., Holappa L. (2003). The effect of silicon, vanadium and nitrogen on the microstructure and hardness of air cooled medium carbon low alloy steels. J. Mater. Process. Technol..

[26] Perrard F., Scott C. (2007). Vanadium precipitation during intercritical annealing in cold rolled TRIP steels. ISIJ Int..

[27] Zackay V.F. (1976). Thermomechanical processing. Mater. Sci. Eng..

[28] Speich G.R., Demarest V.A., Miller R.L. (1981). Formation of Austenite during Intercritical Annealing of Dual-Phase Steels. Metall. Trans. A-Phys. Metall. Mater. Sci..

[29] Zhang Y.J., Chandiran E., Dong H.K., Kamikawa N., Miyamoto G., Furuhara T. (2021). Current Understanding of Microstructure and Properties of Micro-Alloyed Low Carbon Steels Strengthened by Interphase Precipitation of Nano-Sized Alloy Carbides: A Review. JOM.

[30] Lai Q., Gouné M., Perlade A., Pardoen T., Jacques P., Bouaziz O., Bréchet Y. (2016). Mechanism of austenite formation from spheroidized microstructure in an intermediate Fe-0.1 C-3.5 Mn steel. Metall. Mater. Trans. A.

[31] Murakami T., Hatano H., Miyamoto G., Furuhara T. (2012). Effects of Ferrite Growth Rate on Interphase Boundary Precipitation in V Microalloyed Steels. ISIJ Int..

[32] Aristeidakis J.S.S., Haidemenopoulos G.N.N. (2022). Constitutive and transformation kinetics modeling of epsilon-, alpha′-Martensite and mechanical twinning in steels containing austenite. Acta Mater..

[33] Haghdadi N., Cizek P., Beladi H., Hodgson P.D. (2017). A novel high-strain-rate ferrite dynamic softening mechanism facilitated by the interphase in the austenite/ferrite microstructure. Acta Mater..

[34] Haghdadi N., Cizek P., Beladi H., Hodgson P. (2017). Dynamic restoration processes in a 23Cr-6Ni-3Mo duplex stainless steel: Effect of austenite morphology and interface characteristics. Metall. Mater. Trans. A.

[35] Li Z., Wang Y.C., Cheng X.W., Liang J.X., Li S.K. (2020). Compressive behavior of a Fe-Mn-Al-C lightweight steel at different strain rates. Mater. Sci. Eng. A.

[36] Sun B.H., Fazeli F., Scott C., Brodusch N., Gauvin R., Yue S. (2018). The influence of silicon additions on the deformation behavior of austenite-ferrite duplex medium manganese steels. Acta Mater..

[37] Lee S.J., Kim J., Kane S.N., De Cooman B.C. (2011). On the origin of dynamic strain aging in twinning-induced plasticity steels. Acta Mater..

[38] Koyama M., Sawaguchi T., Tsuzaki K. (2018). Overview of Dynamic Strain Aging and Associated Phenomena in Fe-Mn-C Austenitic Steels. ISIJ Int..

[39] Magagnosc D.J., Field D.M., Meredith C.S., An K., Walter T.R., Limmer K.R., Lloyd J.T. (2022). Temperature and stress dependent twinning behavior in a fully austenitic medium-Mn steel. Acta Mater..

[40] Park D.B., Huh M.Y., Shim J.H., Suh J.Y., Lee K.H., Jung W.S. (2013). Strengthening mechanism of hot rolled Ti and Nb microalloyed HSLA steels containing Mo and W with various coiling temperature. Mater. Sci. Eng. A.

[41] Yong Q.L. (2006). The Second Phase of Steel and Iron Material.

[42] Bailey J., Hirsch P. (1960). The dislocation distribution, flow stress, and stored energy in cold-worked polycrystalline silver. Philos. Mag..

